# Correction: Boron-doped few-layer graphene nanosheet gas sensor for enhanced ammonia sensing at room temperature

**DOI:** 10.1039/d0ra90100h

**Published:** 2020-09-30

**Authors:** Shubhda Srivastava, Shubhendra K. Jain, Govind Gupta, T. D. Senguttuvan, Bipin Kumar Gupta

**Affiliations:** CSIR-National Physical Laboratory Dr K S Krishnan Road New Delhi 110012 India tdsen@nplindia.org bipinbhu@yahoo.com; Academy of Scientific and Innovative Research (AcSIR) Ghaziabad-201002 India

## Abstract

Correction for ‘Boron-doped few-layer graphene nanosheet gas sensor for enhanced ammonia sensing at room temperature’ by Shubhda Srivastava *et al.*, *RSC Adv.*, 2020, **10**, 1007–1014. DOI: 10.1039/C9RA08707A

The authors regret that affiliation b was incorrectly provided as Academy of Scientific and Innovative Research (AcSIR), CSIR-National Physical Laboratory Campus, Dr K S Krishnan Road, New Delhi 110012, India.

The correct details are provided in the Affiliations section of this document.

The Royal Society of Chemistry apologises for these errors and any consequent inconvenience to authors and readers.

## Supplementary Material

